# Characterization and phylogenetic analysis of the complete mitochondrial genome of *Glabracollonia laevigata* (Gastropoda: Trochida: Colloniidae) from East China Sea

**DOI:** 10.1080/23802359.2026.2642519

**Published:** 2026-03-11

**Authors:** Qiong Wu, Peng Xiang, Chun Guang Wang, Bing Peng Xing

**Affiliations:** a Third Institute of Oceanography, Ministry of Natural Resources, Xiamen, China; b College of Life Sciences, Beijing Normal University, Beijing, China

**Keywords:** Mitogenome, phylogenetic relationship, sunken wood

## Abstract

Organic falls play an important role in the evolutionary transition of chemosymbiotic mollusks from shallow to deep seas. Exploring their genetic characteristics is essential to elucidating adaptive mechanisms. Here, we report the first complete mitochondrial genome of *Glabracollonia laevigata* (Family Colloniidae), collected from sunken wood at 200 m depth in the East China Sea. The circular genome is 18,056 bp in length with an average sequencing depth of 6847×, and contains 13 protein-coding genes (PCGs), two rRNA genes, and 23 tRNA genes, including a duplicated trnY. The nucleotide composition is biased toward A + T (69.0%). Most PCGs use ATG as the start codon, except nad6 (GTG) and nad4L (ATA), and all terminate with TAA or TAG. Codon usage analysis revealed a preference for A and U at the third codon position. Phylogenetic analyses based on concatenated PCGs, using Bayesian inference and maximum likelihood approaches, strongly support a close relationship between Colloniidae and Phasianellidae, consistent with recent studies. These findings offer new insights into the phylogenetic placement of Colloniidae within Trochoidea, while contributing to a broader understanding of biodiversity and evolutionary adaptation in organic-fall ecosystems.

## Introduction

The family Colloniidae comprises small-sized gastropods that have long been regarded as closely related to the generally larger Turbinidae (Poppe et al. 2023). In some earlier studies, Colloniidae was treated as a subfamily within Turbinidae sensu lato (Williams and Ozawa 2006). Under the classification framework proposed by Bouchet et al. (2017), Colloniidae, together with Turbinidae and eleven other living families, is placed within the superfamily Trochoidea. More recent molecular evidence has further clarified the internal phylogenetic relationships within Trochoidea, consistently indicating that Colloniidae, Phasianellidae, and Areneidae form a distinct clade within the superfamily (Williams and Ozawa 2006; Gong et al. 2025).

Species of family Colloniidae are relatively common on sunken wood in the East China Sea. Studies on Colloniidae are valuable for improving our understanding of organic-fall ecosystems, which play an important role in shaping biodiversity and evolutionary processes in deep-sea chemosynthetic ecosystems. However, to date, only two mitochondrial genome sequences of Colloniidae species are available in GenBank—including those deposited in the present study—and none of these mitochondrial genomes has been formally described in a peer-reviewed publication. In this study, we present the first mitochondrial genome of *Glabracollonia laevigata* (G. B. Sowerby III, 1914), providing novel genomic resources that will facilitate future research on the phylogeny and evolutionary adaptation of organic-fall organisms.

## Materials and methods

**Figure 1.**
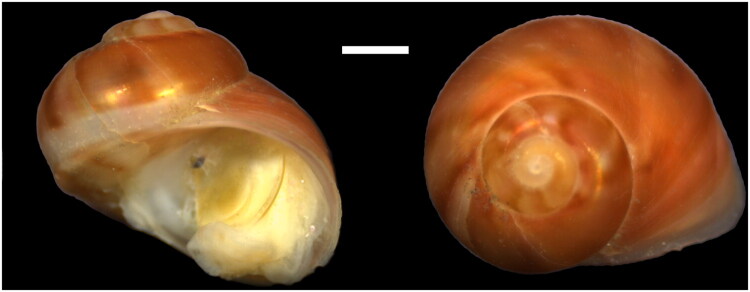
Specimen photographs of *Glabracollonia laevigata*. Scale bar = 1 mm. Photos by Qiong Wu.

A specimen of *Glabracollonia laevigata* was collected from sunken wood at a depth of 200 m in the East China Sea using a trawl vessel (26°20′N, 123°50′E), and subsequently preserved at −60 °C (Figure 1). The species was identified by Huang Shih I based on morphological characteristics. The voucher specimen is deposited in the specimen repository of the Third Institute of Oceanography, Ministry of Natural Resources, under accession number TIO202410DHSW01G1 (specimen contact: Mrs. Yang, Yan Yan, yangyanyan@tio.org.cn).

Total genomic DNA was extracted from foot muscle tissue using the Qiagen DNeasy Blood & Tissue Kit following the manufacturer’s protocol.

For next-generation sequencing, total genomic DNA was sent to Novogene Co., Ltd. (Beijing, China) for library preparation and high-throughput sequencing. Libraries were constructed with an average insert size of approximately 300 bp and sequenced on the Illumina PE150 platform, producing 150 bp paired-end reads. The quality of raw sequencing reads was assessed using FastQC (Andrews 2010). The mitochondrial genome was assembled and annotated with MitoZ (Meng et al. 2019). Protein-coding gene (PCG) boundaries were verified by comparison with closely related species, while tRNA genes were corrected using tRNAscan-SE (Chan et al. 2021). GC content and GC skew were calculated, and the circular genome map was generated using Proksee (Grant et al. 2023).

For phylogenetic reconstruction, we selected 15 species of Trochoidea from GenBank, together with the newly generated *G. laevigata* sequence obtained in this study. Species were selected based on the criterion that each included taxon was represented by at least two mitochondrial genome sequences in GenBank, allowing verification of species identification accuracy. In addition to these ingroup taxa, one mitochondrial genome of *Haliotis virginea* Gmelin, 1791 was included as the outgroup. For each species, the 13 PCGs were concatenated for phylogenetic reconstruction. Multiple sequence alignments were performed using MAFFT (Katoh and Standley 2013) with codon alignment mode, and ambiguously aligned regions were trimmed with Gblocks (Talavera and Castresana 2007). Concatenation was conducted in PhyloSuite (Zhang et al. 2020).

Partitioning schemes and substitution models were selected with ModelFinder (Kalyaanamoorthy et al. 2017) under the Bayesian Information Criterion for both maximum likelihood (ML) and Bayesian inference (BI) analyses. BI trees were inferred in MrBayes v3.2.6 (Ronquist et al. 2012) under partitioned models with two parallel runs of 2,000,000 generations, discarding the first 25% as burn-in. ML analyses were performed in IQ-TREE (Nguyen et al. 2015) under an edge-linked partition model with 2000 ultrafast bootstrap replicates (Minh et al. 2013), the approximate Bayes test (Anisimova et al. 2011), and the Shimodaira–Hasegawa–like approximate likelihood ratio test (Guindon et al. 2010).

The phylogenetic trees were viewed and edited using iTOL (available at https://itol.embl.de/) following (Letunic and Bork 2021).

## Results

**Figure 2.**
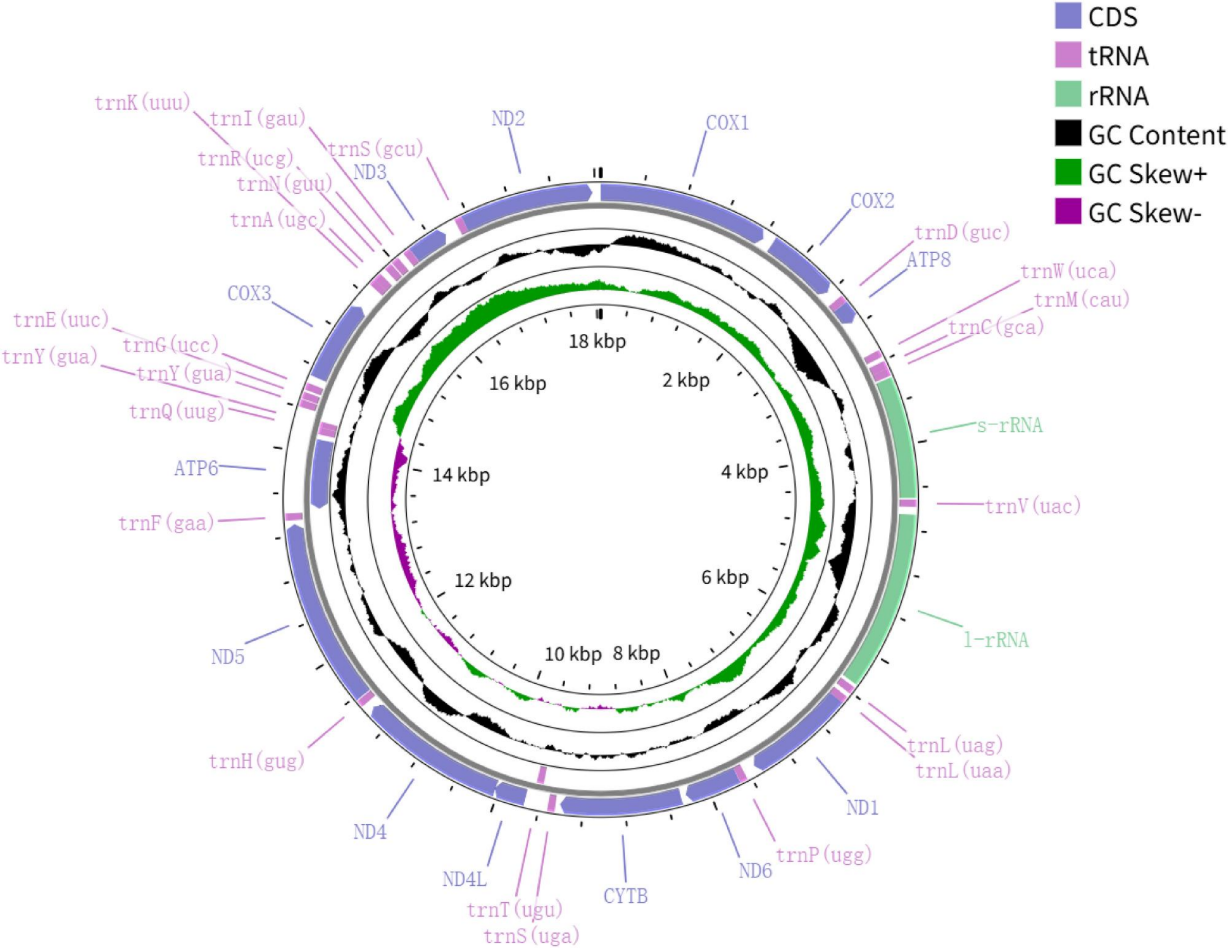
Mitochondrial genome map of *Glabracollonia laevigata*. The circular map depicts protein-coding genes (CDS), tRNAs, rRNAs, GC content, and GC skew, with color-coded annotations indicating functional categories.

The complete mitochondrial genome of *Glabracollonia laevigata* was sequenced at an average depth of 6847× (Suppl. Figure 1). The genome is circular, with a total length of 18,056 bp, and contains 13 protein-coding genes (PCGs), two rRNA genes, and 23 tRNA genes (Figure 2). Compared with typical metazoan mitochondrial genomes, it possesses an additional duplicated trnY (GUA). Except for atp6, the remaining 12 PCGs are located on the heavy strand. The nucleotide composition is A (30.5%), T (38.5%), C (13.8%), and G (17.3%). ATG is the most common start codon, with two exceptions: nad6 initiates with GTG, and nad4L with ATA. All PCGs terminate with either TAA or TAG.

The relative synonymous codon usage (RSCU) analysis revealed a nonrandom codon usage pattern, with a marked preference for A and U at the third codon position (Suppl. Figure 2), indicating a biased usage of synonymous codons.

**Figure 3.**
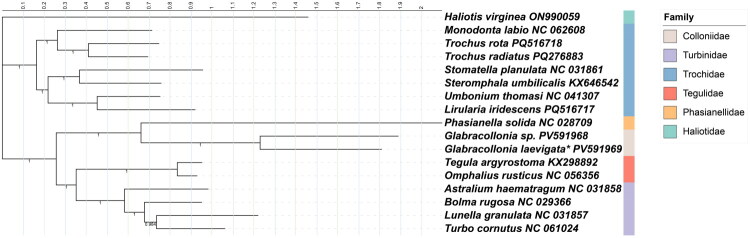
Phylogenetic relationships within the superfamily trochoidea inferred from Bayesian inference (BI) based on 13 mitochondrial protein-coding genes (PCGs). The best-fit substitution models selected by ModelFinder were GTR+F + I + G4 and HKY+F + I + G4. Node values represent Bayesian posterior probabilities. Colored blocks to the right of taxon names indicate family-level classification. The mitogenome newly sequenced in this study were indicated with asterisk mark. All sequences used in the tree were obtained from GenBank, with their corresponding accession numbers and references as follows: ON990059 (Walton et al. 2023), NC_031861 (Lee et al. 2016), KX646542 (Wort et al. 2017), NC_041307 (Kim et al. 2019), PQ516717 (unpublished), NC_062608 (unpublished), PQ516718 (unpublished), PQ276883 (unpublished), NC_028709 (Uribe et al. 2016), PV591968 (unpublished), PV591969 (this study), KX298892 (Lee et al. 2016), NC_056356 (Mao et al. 2020), NC_031858 (Lee et al. 2016), NC_029366 (Uribe et al. 2016), NC_061024 (Kim et al. 2022), NC_031857 (Lee et al. 2016).

Phylogenetic trees reconstructed using both approaches recovered two major lineages with strong overall support. Trochidae formed a distinct clade, whereas the remaining four families—Colloniidae, Turbinidae, Phasianellidae, and Tegulidae—constituted a separate evolutionary lineage. Within Trochidae, the internal relationships were not consistently supported between the two phylogenetic reconstructions. In contrast, the topological structure of the second lineage was congruent across both trees, with strong support for a closer relationship between Colloniidae and Phasianellidae, rather than Turbinidae (Figure 3, Suppl. Figure 3).

## Discussion and conclusions

According to WoRMS (Cuvelier 2005), the superfamily Trochoidea comprises 13 families. Traditionally, Colloniidae has been considered closely related to Turbinidae (Poppe et al. 2023). However, our findings are consistent with Gong et al. (2025), supporting a sister-group relationship between Colloniidae and Phasianellidae.

As of October 2025, only two mitochondrial genomes of Colloniidae species are available in GenBank, including the one generated in this study. Consequently, it is not yet possible to reconstruct detailed phylogenetic relationships within the family. Nevertheless, our analyses successfully resolved relationships within the superfamily Trochoidea. The reconstructed phylogeny included five families—Colloniidae, Phasianellidae, Tegulidae, Turbinidae, and Trochidae—and revealed that Colloniidae, Phasianellidae, Tegulidae, and Turbinidae form a well-supported clade, with Trochidae as their sister group.

This study first formally describe complete mitochondrial genome of *Glabracollonia laevigata*. The genomic data generated here not only contribute to clarifying the phylogenetic position of the family Colloniidae within the superfamily Trochoidea but also provide a valuable resource for future investigations into the biodiversity of sunken wood communities.

## Supplementary Material

SupplFig1.jpg

SupplFig2.jpg

Figure S3.jpg

## Data Availability

The data supporting the findings of this study are publicly available in the NCBI GenBank database (https://www.ncbi.nlm.nih.gov) under the BioProject PRJNA1149176, with accession number PV591969, BioSample ID SAMN52353653 and SRA ID SRR35730924.
