# Supplementary figures and images for: Characterization and phylogenetic analysis of the complete mitochondrial genome of Glabracollonia laevigata (Gastropoda: Trochida: Colloniidae) from East China Sea
Source: Mitochondrial DNA B Resour. 2026 Mar 11;11(4):536–40. doi: 10.1080/23802359.2026.2642519 (PMC12981266; doi:10.1080/23802359.2026.2642519)

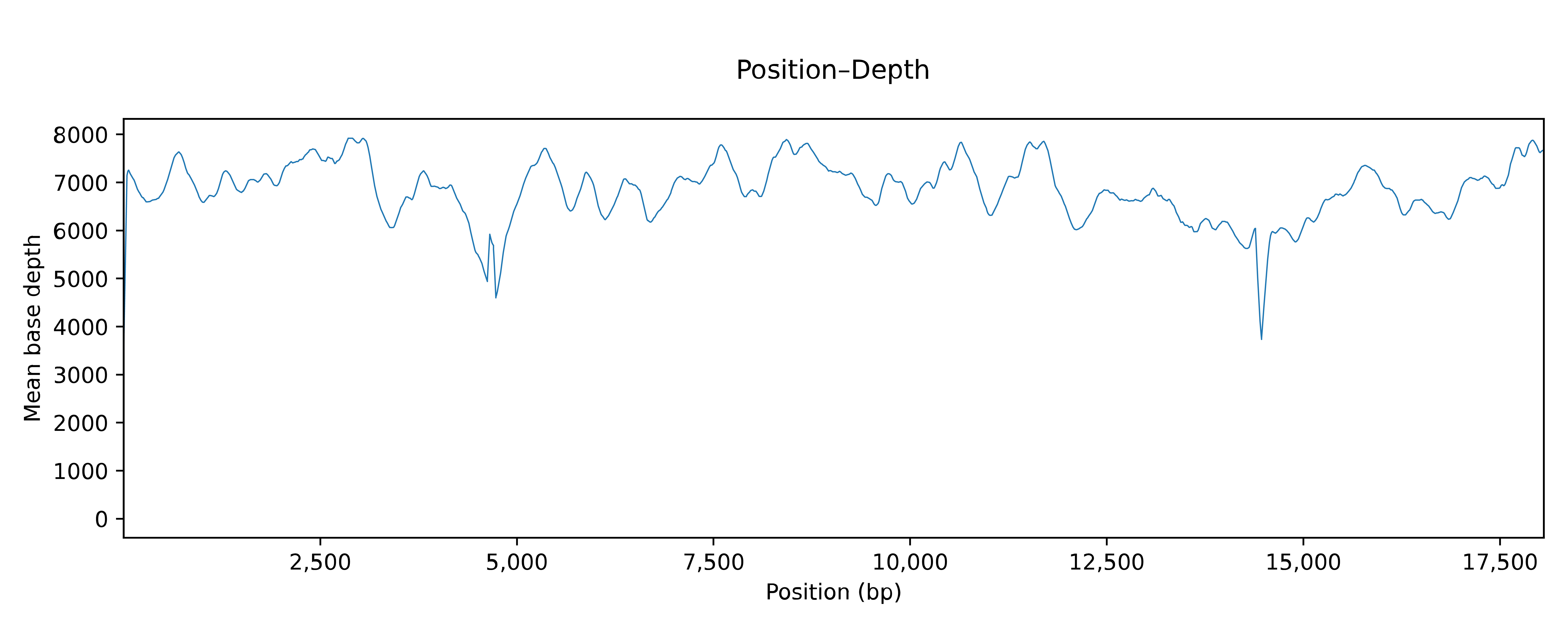

Supplement: SupplFig1.jpg [file TMDN_A_2642519_SM9589.jpg]

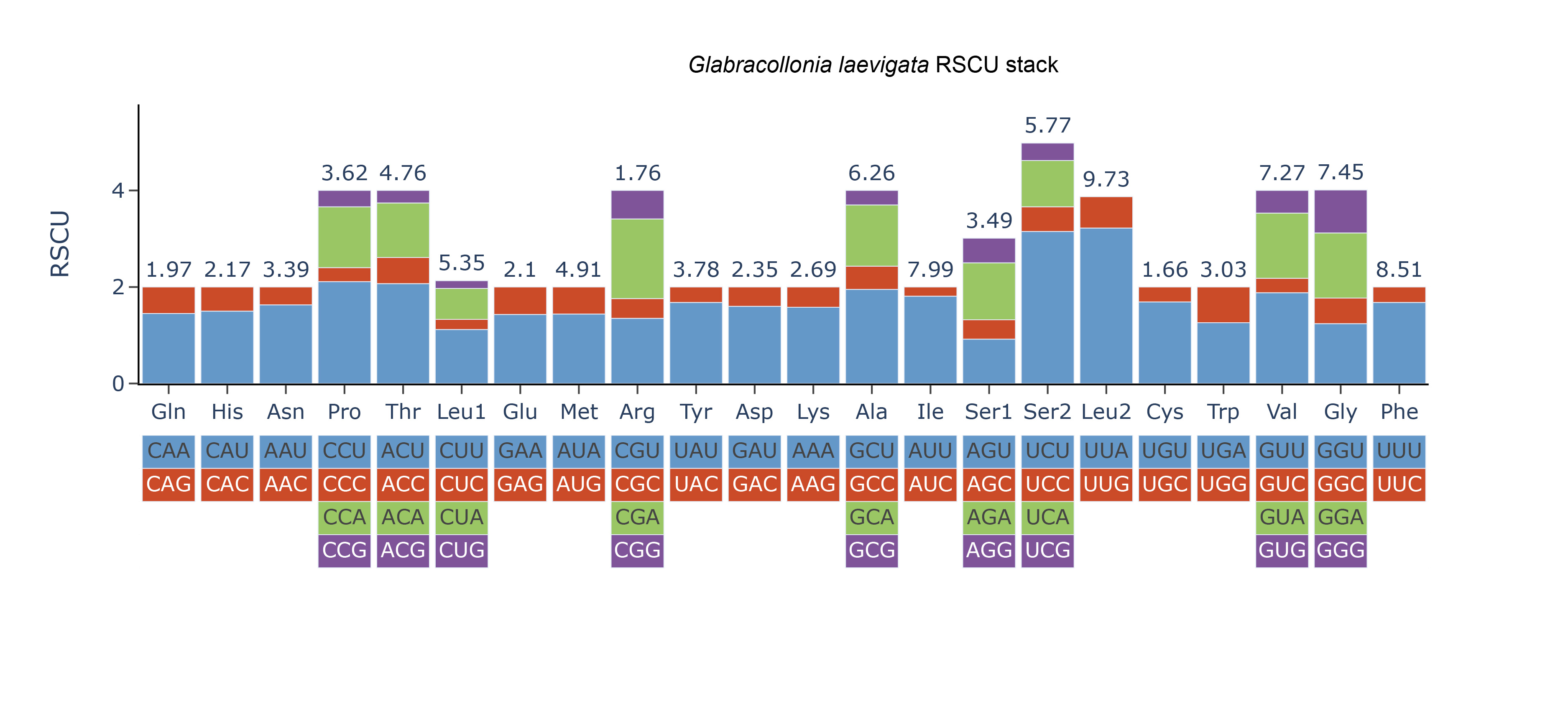

Supplement: SupplFig2.jpg [file TMDN_A_2642519_SM9588.jpg]

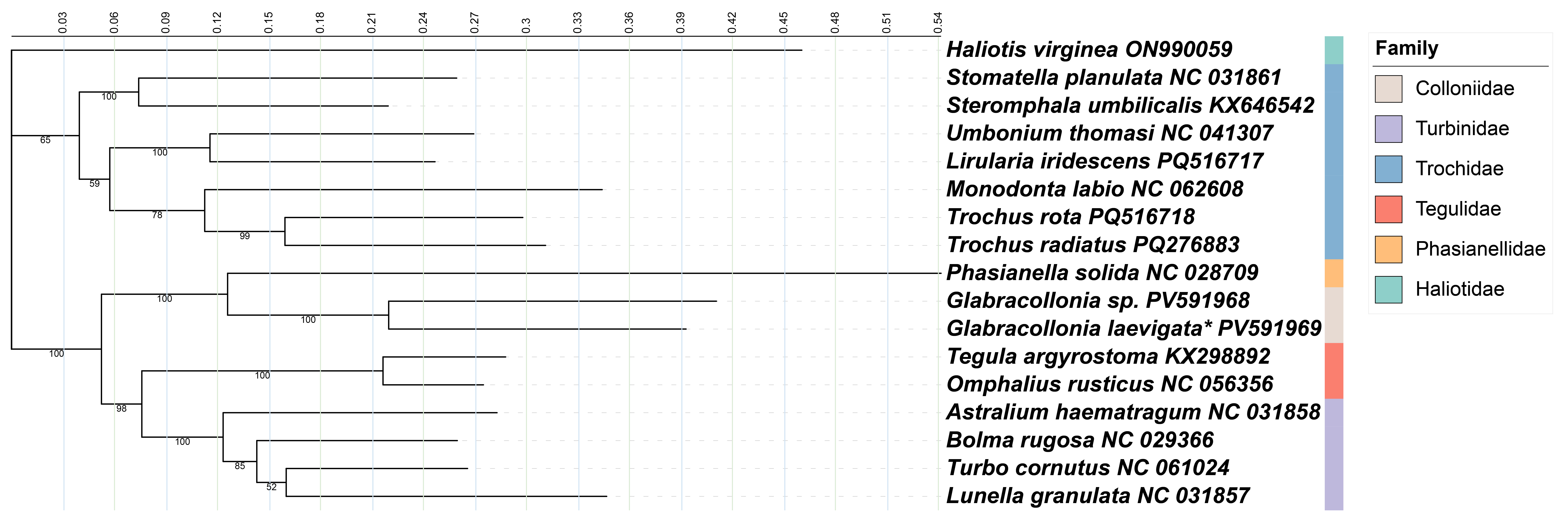

Supplement: Figure S3.jpg [file TMDN_A_2642519_SM9587.jpg]
